# Comparison of activity and fatigue of the respiratory muscles and pulmonary characteristics between post-polio patients and controls: A pilot study

**DOI:** 10.1371/journal.pone.0182036

**Published:** 2017-07-27

**Authors:** David Shoseyov, Tali Cohen-Kaufman, Isabella Schwartz, Sigal Portnoy

**Affiliations:** 1 Pediatric department, Hadassah Mount Scopus, Jerusalem, Israel; 2 Physical Medicine and Rehabilitation department, Hadassah Mount Scopus, Jerusalem, Israel; 3 Department of Physiotherapy, Sackler Faculty of Medicine, Tel Aviv University, Tel Aviv, Israel; 4 Department of Occupational Therapy, Sackler Faculty of Medicine, Tel Aviv University, Tel Aviv, Israel; National Yang-Ming University, TAIWAN

## Abstract

**Objectives:**

To compare pulmonary function measures, maximal respiratory pressure and fatigue of respiratory muscles between patients with Post-Polio Syndrome (PPS) and controls.

**Design:**

Cross-sectional study.

**Patients:**

Patients with PPS (N = 12; age 62.1±11.6 years) able to walk for 6 minutes without human assistance; age-matched controls with no history of polio or pulmonary dysfunction (N = 12; age 62.2±6.5 years).

**Measurements:**

A body plethysmograph was used to quantify Residual Volume (RV), Total Lung Capacity (TLC), and Thoracic Gas Volume (TGV) etc. A manometer was used to measure Maximal Inspiratory Pressure (MIP) and Maximal Expiratory Pressure. A spirometer was used to measure Maximal Voluntary Ventilation (MVV). Surface electromyography (sEMG) recorded diaphragmatic muscle activity while performing MVV.

**Results:**

The control group had significantly higher TGV and showed improvement in MIP following the effort (difference of 5.5±4.0cmH_2_O) while the PPS group showed deterioration in MIP (difference of -2.5±5.0cmH_2_O). Subjects with scoliosis had significantly higher RV/TLC values compared with subjects without scoliosis. The 25^th^ frequency percentile of the sEMG signal acquired during MVV was reduced in the PPS group.

**Conclusions:**

Maximal respiratory pressure test and sEMG measurements may identify fatigue of respiratory muscles in patients with PPS. Early diagnosis of respiratory impairment may delay respiratory decline and future need of invasive respiratory aids.

## 1. Introduction

Post-Polio Syndrome (PPS) was described 15 years following the first outbreak of Poliomyelitis, when new neuromuscular symptoms were reported. PPS is reported to develop in 20–75% of polio survivors, 15 years or more after acute disease [1–10]. Possible risk factors for PPS are age, genetics, time since the acute poliomyelitis, stress and inactive lifestyle [11]. PPS symptoms mainly comprised of new muscle weakness or deterioration of previously affected muscles, combined with muscle atrophy, fatigue, muscle and joint pain and overall functional deterioration [12]. Although good-quality data regarding the efficacy of different PPS interventions are scarce [13], new muscle weakness is frequently treated with non-fatiguing exercise which avoids muscle overuse [14]. Additionally, lower extremity orthoses, assistive devices, physical therapy and pharmacologic agents may be used [14].

An additional symptom of PPS and the main cause of death during acute polio is respiratory disorder. Although respiratory insufficiency is usually caused by weakness of respiratory muscles or bulbar muscle dysfunction, it is also related to the high incidence of secondary complications, e.g. scoliosis [15], obesity [16], and sleep disordered breathing (sleep apnea) in individuals with PPS [17]. According to pathophysiology, survivors of polio mainly suffer from restrictive respiratory impairment, rather than other causes. Currently, approximately 27–36% of the survivors of polio suffer from respiratory insufficiency [18]. The main respiratory complaints are dyspnea, fatigue, or sleep-related disordered breathing [19]. While the aforementioned PPS symptoms are not life-threatening, respiratory disorder raises the risk of morbidity and mortality [17,20].

Early diagnosis of respiratory impairment and introduction to non-invasive ventilatory aids may delay respiratory decline and future need of invasive respiratory aids [21]. The diagnosis of pulmonary dysfunction can be performed using several pulmonary tests, regularly used in the clinical setting. The restrictive pulmonary impairment of patients with PPS is characterized by Total Lung Capacity (TLC) value below the 5^th^ percentile of the predicted value [22]. It may first be suspected when the Vital Capacity (VC) is reduced; however a reduced VC by itself might be related to either restrictive or obstructive ventilatory defect [22]. In an 11-year long prospective study of 31 patients with PPS, more than half of the patients with a VC below 50% developed a need for ventilation or suffered respiratory failure related death [23]. The low VC measurements however were not associated with ventilation during the acute stages of the illness [23]. Also, patients with PPS that had scoliosis showed a lower VC [23]. The authors suggested that early determination of VC may be useful in identifying high-risk patients with PPS. Another prevalent method for studying respiratory muscle fatigue is to measure of Maximal Voluntary Ventilation (MVV) which is an objective dynamic method for measuring the working capacity of respiratory muscles, i.e. muscle endurance [22,24]. Respiratory muscle fatigue may induce dyspnea and CO_2_ retention [25]. A previous study [26] reported significantly lower MVV in PPS patients compared to controls but no difference compared to polio survivors. The three studied groups were small (9–10 subjects per group) and mostly in their late 40s or early 50s.

The aforementioned tests may not be sensitive enough for detection of the early stages of inspiratory deterioration in PPS patients. A more objective, quantitative method for evaluation respiratory muscle fatigue may therefore be warranted, and this was the rational for the present study.

The possible etiology and diagnosis of PPS symptoms may be further evaluated by means of ElectroMyoGraphy (EMG), single fiber EMG (SFEMG), and macro-EMG. Muscular fatigue might be caused by neuromuscular junction transmission defects [27]. Muscle weakness and atrophy might be related to distal degeneration of motor units, resulting in irreversible muscle fiber denervation [27]. Currently, surface EMG (sEMG) has been documented as a potential alternative for needle EMG or nerve conduction studies when investigating of neuromuscular disorders [28]. This method is advantageous due to its non-invasiveness and large signal detection area. In an evidence-based review of the use of sEMG in the diagnosis and study of neuromuscular disorders [28], the authors suggest that sEMG may provide additional information in the study of fatigue in patients with PPS. An example of this was shown in [29] where high density sEMG measurements of the quadriceps activity were used to discriminate between healthy subjects and subjects with PPS. In a recent example, sEMG of the abdominal muscles was used to compare a population at risk for Chronic Obstructive Pulmonary Disease (COPD) with healthy subjects [30]. The research group had a different recruitment pattern of abdominal muscles for the mechanics of breathing. However, the two groups showed significant differences in FEV1, peak expiratory flow and forced expiratory flow, meaning that detection of inspiratory impairment was possible without the EMG. To our knowledge, no previous study performed a comprehensive measurement battery that includes the following parameters: MVV, Maximal Inspiratory Pressure (MIP), Maximal Expiratory Pressure (MEP), recordings of respiratory muscle fatigue, and pulmonary function tests in patients with PPS. Specifically, no data regarding respiratory muscle fatigue in patients with PPS has been reported in the literature.

The aims of this study were therefore (i) to compare pulmonary function measures and maximal respiratory pressure between patients with PPS and control subjects and (ii) to compare activity levels and fatigue of respiratory muscles between patients with PPS versus control subjects, using sEMG following a supervised respiratory effort.

## 2. Methods

### 2.1 Setting and participants

For this cross-sectional study, we recruited 12 patients with PPS, registered for PPS clinic consultation in the PPS center at the Hadassah physical medicine and rehabilitation department in Jerusalem. Recruitment date was January 2013. We included patients diagnosed with PPS, according to published criteria [31], able to walk for 6 minutes, with or without walking aids and orthotics but without human assistance. Twelve subjects with no history of polio or pulmonary dysfunction were recruited as an age-matched control group. See Table 1 for subject characteristics. The study was approved by the Helsinki committee of the hospital (approval #0534-13-HMO) and registered in clinicaltrials.gov (NCT03064711).

**Table 1:** Personal characteristics of the Post Polio Syndrome (PPS) group and the control group. Numeric values are presented as mean ± standard deviation. Other data are presented as the number of subjects (percent of each group; N = 12).

	PPS (N = 12)	Control (N = 12)	P value
Gender (M = Male; F = Female)	6M, 6F	4M, 8F	.680^¥^
Age (years)	62.1 ± 11.6	62.2 ± 6.5	.772^ξ^
	Range 40 to 81	Range 45 to 70	
Height (cm)	159.9 ± 10.2	165.2 ± 9.8	.198^ξ^
Weight (kg)	78.5 ± 13.8	70.5 ± 14.2	.143^ξ^
Age of initial infection with the virus (years)	2.5 ± 3.2	-	
Years since PPS diagnosis	13.7 ± 7.6	-	
Neurologic deterioration in the last 2 years	6 (50%)	-	
Respiration difficulty during acute polio	1 (8.3%)	-	
Difficulty breathing	9 (75%)	-	
Sleep apnea	4 (33.3%)	1 (8.3%)	
Scoliosis	7 (58.3%)	-	
Smoking	1 (8.3%)	0 (0%)	

^¥^Chi square test;

^ξ^Mann-Whitney test.

### 2.2 Research tools

A body plethysmograph (ZAN 500W, nSpire Health Ltd, United Kingdom) was used to quantify the following: Forced Expiratory Volume in the first second of a forced expiratory maneuver initiated at total lung capacity (FEV1), VC, Slow Vital Capacity (SVC), Residual Volume (RV), Total Lung Capacity (TLC), Thoracic Gas Volume (TGV). Also, RV to TLC ratio is calculated, as well as FEV1 to CV ratio. These measurements are provided in liters and presented as percentage of the predicted normal values according to data of the same age, gender, height or arm span (for scoliosis patients) and weight [32]. We used a hand-held portable, digital manometer (MicroRPM, Carefusio) to measure MIP and MEP in cmH_2_O and normalize to predicted values, according to the following equations [33]: for males, 0.158∙BMI-0.052∙age+8.22 and for females: 8.55–0.024∙age.

These values represent the strength of the inspiratory and expiratory muscles, correspondingly [34]. Values are measured during one second with a resolution of 1cmH_2_O with accuracy of 3%. A spirometer (KOKO Pulmonary Function Test Diagnostic Spirometer, Version 3, Germany) was used to measure MVV which induces diaphragm fatigue [35].

**Fig 1:**
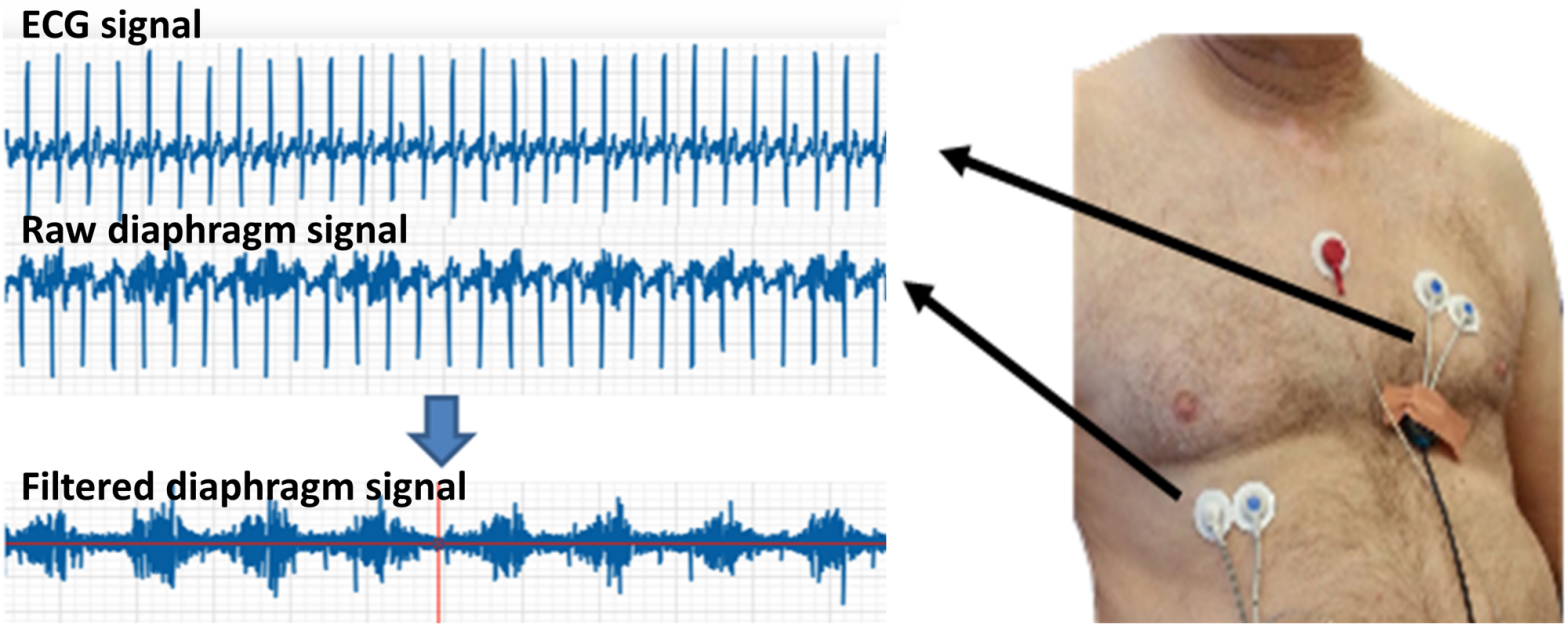
Placement of the surface electrodes on the thoracic muscles and above the heart of a healthy male subject (right frame) and the recorded heart activity signal subtracted from the raw diaphragm signal to produce the diaphragm activity signal.

Finally, an 8-channel Bluetooth telemetric surface electromyography (EMG) system (Zebris Medical GmbH, Germany) was used to record the right costal diaphragmatic muscle activity during rest and while performing MVV. Disc-shaped bipolar electrodes (diameter of the electrode disposable sticker is 26mm and its gel-filled sensing diameter is 9mm) were used. The common mode rejection ratio is 110dB with an amplification of 1000. Data were recorded at a frequency of 1500Hz. After the skin was cleaned, the electrodes were attached according to a validated protocol [36,37]. Specifically, the electrodes were placed by a trained physiotherapist (TCK) at the seventh or eighth intercostal space on the right side of the body at the midclavicular line in the orientation of the muscle fibers. A ground electrode was placed on the sternum. A clear EMG signal during deep inspiration was confirmed. Two additional electrodes were placed on the left side of the thorax in a Lead II configuration so that the ECG signals could be subtracted from the recordings of the activity of the diaphragm (Fig 1). These electrodes were also used to compute the heart rate using the R-R distances of the QRS complexes.

### 2.3 Protocol

Each subject read and signed an informed consent form pretrial. Then the subject filled out a demographic and medical history questionnaire (S1 File) and was measured for height and weight. The arm span length of the PPS group was also measured. The subject was then provided with a nose clip and sat in the plethysmograph chamber and performed the examination for lung volumes. After approximately five minutes of rest, the subject held a digital manometer and was requested to fully exhale and then inspire maximally once a tight seal was created around the mouth manometer for measurements of MIP and MEP. The subject repeated this three times with at least 30 seconds of rest between each repetition in order to prevent testing-induced respiratory muscle fatigue. The electrodes were then placed on the subject according to the aforementioned protocol and a 30 seconds duration of quiet breathing was recorded. Then the spirometer was used to perform MVV maneuvers, during which, the subject was requested to sit upright to prevent restriction of airflow. The subject breathed deeply and rapidly for 20 seconds, then rested 20 seconds and repeated the MVV. The EMG signals were recorded continuously during the entire MVV trial. Following a 5 minute rest, the subject repeated the MIP and MEP measurement with the digital manometer.

### 2.4 Post analysis

Statistical analyses were performed using SPSS version 21. Data are provided in S2 File. Shapiro-Wilk test was used to check normality of data distribution. Grubb’s test was used to detect outliers. The following parameters were found to be normally-distributed and therefore their descriptive data are presented as mean and Standard Deviation (SD) and differences in and between groups were tested using 2-tails paired and unpaired student’s t-test, respectively: TGV, TLC, VC, RV, MVV, all MIP and MEP except MEP before MVV, RMS and the quartile percentiles of sEMG frequency. The SVC, FEV1, RV/TLC, and MEP before MVV were not normally-distributed and therefore their descriptive data are presented as median and Interquartile Percentiles (IQR) and differences in and between groups were tested using Wilcoxon signed-rank test and Mann-Whitney U test, respectively. Apart from the analyses performed for this pilot study aims, we investigated whether there was a difference in the aforementioned measures in the group with PPS alone, between patients with and without scoliosis and neurologic deterioration in the last 2 years. Due to the small sample size, these analyses were added after the data collection when it was evident that there were approximately 50% of the subjects in each group. Finally, analyses in the PPS group alone were performed using the Mann-Whitney U test due to the small sizes of the groups divided by factors as scoliosis and neurologic deterioration in the last 2 years. P-values of less than 0.05 were considered to be statistically significant.

## 3. Results

There were no significant differences between the PPS and control groups in terms of gender, age, height and weight (Table 1). No between-group differences were found in values of FEV_1_, SVC, TLC, VC, and RV (Table 2). The PPS group had significantly lower TGV compared with the control group, so that nine PPS subjects (75%) had TGV values below 80%. The measured values of SVC and FEV_1_ were normal. Five subjects in each group (41.7%) exhibited FEV_1_ values of 100% or above it.

**Table 2:** Pulmonary function test results in the Post Polio Syndrome (PPS) group (N = 12) and the control group (N = 12) presented as percent of predicted value. Values are presented as mean ± standard deviation.

	PPS	Control	P value
Forced expiratory volume in 1 second (FEV1; %)*	93.5(82.0–108.5)	95.0(85.0–106.0)	.713
FEV1/Vital Capacity (FEV1/VC%)	91.7±8.0	98.5±12.0	.124
Slow vital capacity (%)*	95.0(88.0–107.3)	98.0(90.0–100.0)	.806
**Thoracic gas volume (TGV, %)**	**68.6±28.9**	**119.5±36.4**	**.001**
Total lung capacity (TLC, %)	108.7±33.6	96.7±24.3	.342
Residual volume (RV, %)	121.3±52.9	105.9±46.4	.486
RV/TLC (%)*	101.4(100.0–110.6)	108.1(91.3–114.0)	.803

*Data presented as median and interquartile percentile due to non-normal distribution. Mann-Whitney test was used.

In the PPS group, seven subjects (58.3%) had RV values above 120% which means hyperinflation. Except for the RV/TLC measurement, no differences in all the measured parameters were found between subjects with PPS with (n = 7) and without (n = 5) scoliosis. A significant difference (p = .034) was found between the RV/TLC values of subjects with PPS with (n = 7) and without (n = 5) scoliosis. The median (IQR) values of the RV/TLC were 110.2% (104.2–136.9%) for the PPS subjects with scoliosis and 100.0% (100.0–101.4%) for the PPS subjects without scoliosis.

There were no differences in all the measured parameters between subjects with PPS who showed neurologic deterioration in the last 2 years (n = 6) and subjects with PPS who did not show neurologic deterioration in the last 2 years (n = 6). Although PPS is a progressive syndrome, the deterioration in neurologic symptoms may plateau for up to 10 years [38].

There were not statistical differences between groups in the MVV parameters (Table 3). This is not surprising as we intentionally recruited PPS patients with a certain degree of functionality, i.e. able to walk for 6 minutes. The PPS group did not comprise of wheelchair-bound patients, whose pulmonary function tests may easily point to pulmonary dysfunction. Accordingly, most of our results show that their breathing difficulties are not diagnosed using most custom clinical examinations, measured using a body plethysmograph. Both groups showed median values lower than normal (<80%). In the PPS group, 8 subjects (66.7%) had values lower than 80% while in the control group there were only 4 subjects (36.4%) with values below 80%. These results may not alert to a pathology. A statistically significant negative correlation exists between age and MVV. Most of our control subjects were in their 60s. Three of the four controls with MVV<80% were females, for which normal MVV values are expected to be 95.7±19.3% [39], so the values below 80% are normal. Since MVV is influenced by respiratory muscles strength, as well as by the compliance of the lung-thorax system, the state of the ventilatory control systems and the resistance of the airways and soft tissues. Aging is associated with reduction in respiratory muscles strength, a reduction in the compliance of the chest wall, and an increase in the resistive and elastic work of breathing.

**Table 3:** Maximal Voluntary Ventilation (MVV), Maximal Inspiratory Pressure (MIP), and Maximal Expiratory Pressure (MEP) in the Post Polio Syndrome (PPS) subjects (N = 12) and the heathy subjects (N = 12), presented as percent of predicted value. Values are presented as mean ± standard deviation.

		PPS	Control	P value
MVV	(%)	73±27	77±24	.848
MIP	Before MVV (%)	75±30	61±29	.261
	Before MVV (cmH2O)	68±27	55±28	.255
	After MVV (%)	72±29	67±27	.687
	After MVV (cmH2O)	66±26	60±27	.645
	**Difference (cmH2O**)	**-2.5±5.0**	**5.5±4.0**	**.001**
MEP	Before MVV (%)*	83(47–114)	71(44–189)	.687
	Before MVV (cmH2O)	77±26	70±32	.577
	After MVV (%)	72±26	83±33	.382
	After MVV (cmH2O)	72±31	73±29	.937
	Difference (cmH2O)	-5(-26-27)	0(-25-42)	.056

*Data presented as median and interquartile percentile due to non-normal distribution.

**Fig 2:**
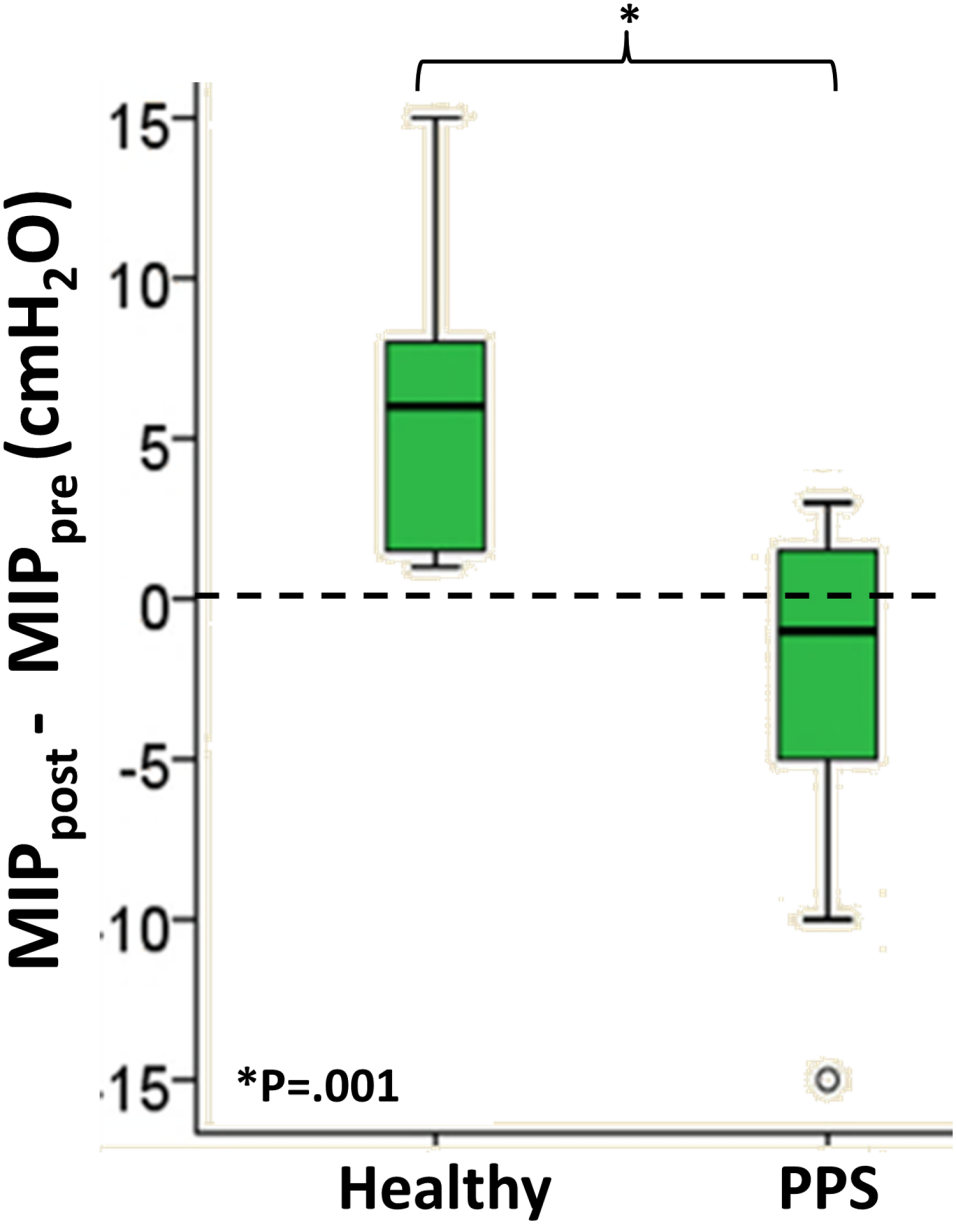
Difference in Maximal Inspiratory Pressure (MIP) before and after Maximal Voluntary Ventilation (MVV) maneuvers in the control and Post Polio Syndrome (PPS) groups.

No statistical differences between groups were found in the measurements of MIP and MEP before and after MVV (Table 3). However, when comparing the difference in MIP after and before MVV, the control group showed improvement in MIP following the effort (difference of 5.5±4.0 cmH_2_O) while the PPS group showed deterioration in MIP following the effort (difference of -2.5±5.0 cmH_2_O). This finding is depicted in Fig 2.

The heart rate of the subjects at rest was 76.4±17.0 and 85.9±10.7 beats per minute for the control and PPS groups, respectively. The heart rate was elevated during the MVV maneuvers to 94.0±15.3 and 100.8±8.1 beats per minute for the control and PPS groups, respectively.

There were no significant differences in the sEMG parameters of RMS and frequency analysis but for the reduction in the 25^th^ frequency percentile in the PPS group (Table 4). Both groups showed a trend towards muscle fatigue. The only subject that received respiratory support in acute polio had a median frequency at the end of MVV that was two thirds lower than the average median frequency of the PPS group, i.e. her median frequency was reduced by 8.9Hz while the average reduction in the PPS group was 3.1Hz.

**Table 4:** Root mean square (RMS) and frequency quartiles (25, 50, and 75) 0f the electromyography (EMG) signal acquired during Maximal Voluntary Ventilation (MVV) in the Post Polio Syndrome (PPS) group and the control group. Values are presented as mean ± standard deviation.

	Beginning of MVV		End of MVV	
	PPS	Control	PPS	Control
RMS (mV)	10.9±4.1	11.2±7.7	12.1±4.5	11.8±7.8
25 percentile of frequency (Hz)	**44.3±6.3**	41.8±9.4	40.7±4.5*	38.7±7.2
50 percentile of frequency (Hz)	60.2±10.2	58.3±11.9	56.6±8.4	55.3±9.1
75 percentile of frequency (Hz)	78.8±17.0	80.3±16.7	75.5±14.4	75.3±10.9

*p = .028

## 4. Discussion

Respiratory deterioration is a life threatening condition which decreases life expectancy. In this study, we aimed to find, through comprehensive measurement methods, several parameters that could discriminate between the pulmonary characteristics and respiratory muscle activity of patients with PPS and a control group. Early detection of fatigue of respiratory muscles may allow the clinicians to modify the treatment plan so that physiotherapy treatment may enhance muscle endurance [21]. Our main findings show differences between the PPS and control groups in measurements of TGV (Table 2) and the difference in MIP measured before and after MVV maneuvers (Fig 2; Table 3). No significant differences were found in activity levels and fatigue of the respiratory muscles, excluding the 25^th^ frequency percentile in the PPS group (Table 4) or measurements of the pulmonary function test and MVV, excluding MIP difference (Fig 2; Table 3).

Interestingly, the RV/TLC measurement was able to discriminate between subjects with PPS with and without scoliosis. In a study of 44 patients with idiopathic scoliosis and 16 patients with PPS and scoliosis, groups were divided into normal and abnormal pulmonary function subgroups [40]. The abnormal pulmonary function of the scoliotic subjects was mostly restrictive, i.e. decreased VC, increased RV and relatively normal expiratory airflow. The authors reported negative correlation between MIP, MEP, and FVC in both groups and combined features of the spinal deformity, e.g. the scoliotic angle and the degree of rotation of scoliosis at the apex. Additionally, significant differences in RV/TLC were found between the idiopathic and PPS group, as this value was 10% higher in the PPS group.

In this study, no differences in respiratory muscle strength measured by MIP and MEP were found. Similar finding were previously documented in a study that compared healthy subjects with polio group and PPS group [26]. Measurements were not recorded however before and after MVV. Whereas in the present study, the difference between MIP measured before and after MVV was significantly higher in the control group and positive on average (Table 3; Fig 2), showing improvement. This improvement in MIP suggest a learning effect. A previous study in healthy subjects compared MIP before and after a respiratory “warm-up” with a device that requires continuous application of inspiratory pressure to keep a valve open [41]. Following the “warm-up”, there was a significant increase in MIP. When further repeating the test, a plateau was observed. The learning effect in repeated MIP was also documented in 178 subjects showing that MIP, averaged of the first 3 out of 20 highest values with up to 5% variability was significantly smaller than the average of the 3 highest values with up to 5% variability from all recorded maneuvers [42]. Conversely, in the PPS group, the difference was negative, possibly due to lack of additional active motor units that ought to be recruited for the effort. This finding suggests that the test of muscle strength is not sensitive enough to detect pulmonary dysfunction in patients with PPS without performing MVV.

Recording of sEMG signals is an objective and precise method for the evaluation of neuromuscular abnormalities [28,43]. However, most published literature is concerned with muscles of the lower limb and report an increase in RMS signal and decrease in the median frequency following muscle fatigue [44,45]. In this study, no significant differences were found in the RMS and frequency quartiles between groups. Also, in-group differences were not statistically significant, excluding the 25^th^ frequency percentile in the PPS group, although a trend towards frequency quartiles decrease was observed in both groups (Table 4). We assume that our protocol for inducing respiratory muscle effort via MVV maneuvers was insufficient in producing detectable muscle fatigue.

The main limitation of this study was that although MVV is a conventional method to induce pulmonary effort, our protocol might have been too short to induce fatigue in the healthy group. However, the fatigue in respiratory muscles found in the PPS group suggests that this fast measurement protocol, that does not require physical exercise or high respiratory discomfort, may be suitable for early detection of respiratory muscle fatigue in PPS patients. Additionally, the small sample size does not necessarily represent the entire PPS population. Finally, we did not set low MVV values as exclusion criteria for the controls, so that four subjects in the control group had MVV below 80%. However, we tested the outcome measures for outliers inside the control group alone using Grubb’s test, and no outliers were detected, so we assume that the results of these four subjects were not abnormal.

Our results suggest that the maximal respiratory pressure test and sEMG measurements may identify fatigue of respiratory muscles in patients with PPS. These results might be reproducible in polio survivors without PPS. We recommend developing a suitable training program to prevent respiratory complications in these patients in order to avoid the need for respiratory assist devices. Recommendation for future studies include a more intensive task to achieve respiratory muscle fatigue, comparison between patients with PPS that were assisted by a respirator during the acute polio and those that were not and finally, studying the effect of different interventions on RV/TLC and MIP difference before and after MVV.

## Supporting information

S1 File A 20-question demographic and medical history questionnaire. (DOCX)

S2 File The raw data (provided in an SPSS file). (SAV)

## Abbreviations

FEV1: Forced Expiratory Volume in the first second
IQR: Interquartile Percentiles
MEP: Maximal Expiratory Pressure
MIP: Maximal Inspiratory Pressure
MVV: Maximal Voluntary Ventilation
PPS: Post-Polio Syndrome
RV: Residual Volume
SD: Standard Deviation
sEMG: Surface electromyography
SFEMG: single fiber EMG
TLC: Total Lung Capacity
TGV: Thoracic Gas Volume
