# Supplementary material for: Comparison of activity and fatigue of the respiratory muscles and pulmonary characteristics between post-polio patients and controls: A pilot study
Source: PLoS One. 2017 Jul 27;12(7):e0182036. doi: 10.1371/journal.pone.0182036 (PMC5531432; doi:10.1371/journal.pone.0182036)
Supplement: S1 File — (DOCX) [file pone.0182036.s001.docx]

שאלון (קבוצת הביקורת תמלא שאלות 1 עד 9)

1. נבדק מספר: ______

2. גיל: _____

3. מצב משפחתי: ______

4. גובה: ______ 5. משקל: _______

6. מעשן?______

7. האם עובד? ________ 8. מספר שעות עבודה ביום_____

9. האם אופי העבודה הוא יושבני או שרוב העבודה בעמידה? _____

10. באיזה גיל חלית במחלת הפוליו? ____

11. מה היו הסימנים של המחלה הראשונית:

חולשה/ שיתוק:

א. ביד אחת ה. בשתי הידיים וברגל אחת

ב. בשתי הידיים ו. בשתי הרגליים וביד אחת

ג. ברגל אחת ז. בשתי הידיים ובשתי הרגליים

ד. בשתי הרגליים

12. האם קיבלת סיוע נשימתי בזמן התקף המחלה? ______

13. באיזה גיל אובחנת כסובל מתסמונת פוסט פוליו? ______

14. האם אתה סובל מקושי במאמצים עקב קשיי נשימה? _____

15. האם ביצעת בעבר בדיקת תפקודי ריאות/ אובחנת כסובל מבעיה נשימתית? _____

16. במידה ואתה סובל מבעיות נשימה, האם טופלת בעבר או מטופל כיום בפיזיותרפיה נשימתית? _____

17. האם אתה זקוק בלילה לתמיכה נשימתית? _____

18. האם אתה סובל מעקמת גבית? ______

19. האם אתה זקוק בהליכה למכשירי הליכה? (אם כן, איזה?) ______

20. האם אתה זקוק בהליכה לאביזרי עזר כגון קביים? (אם כן, איזה?) ________

Questionnaire (the control group will fill out Q1 to Q9)

1. Subject number: ________

2. Age: __________

3. Marital status:________

4. Height __________ 5. Weight __________

6. Smoking? __________

7. Employed? _________ 8. Work hours per day ______________

9. Is most work performed while sitting or standing? __________

10. How old were you when you first got infected with the polio virus? ___________

11. What were the symptoms of the acute polio?

Weakness / paralysis in:

a) one hand e) both hands and one leg

b) two hands f) both legs and one hand

c) one leg g) both legs and both ands

d) both legs

12. Did you receive respiratory support during acute polio? ________

13. How old were you when you were diagnosed with PPS? _________

14. Do you suffer from difficulty in strenuous activity due to breathing difficulties? _____

15. Have you ever tested/diagnosed for pulmonary dysfunction? _________

16. If so, have you ever been treated or are treated today in respiratory physiotherapy? _______

17. Do you require night respiratory support? _______

18. Do you have scoliosis? ______

19. Do you use walking aids? If so, which ones? ____________

20. Do you use walking devices, like crutches? If so, which ones? ___________
